# Supplementary material for: Collective Cell Behavior in Mechanosensing of Substrate Thickness
Source: Biophys J. 2018 Jun 7;114(11):2743–55. doi: 10.1016/j.bpj.2018.03.037 (PMC6027966; doi:10.1016/j.bpj.2018.03.037)
Supplement: Document S1. Figs. S1–S5 [file mmc1.pdf]

Biophysical Journal, Volume *114*

Supplemental Information

Collective Cell Behavior in Mechanosensing of Substrate Thickness

Camelia G. Tusan, Yu-Hin Man, Hoda Zarkoob, David A. Johnston, Orestis G. Andriotis, Philipp J. Thurner, Shoufeng Yang, Edward A. Sander, Eileen Gentleman, Bram G. Sengers, and Nicholas D. Evans

SUPPLEMENTARY INFORMATION FOR “*Collective cell behaviour in mechanosensing of substrate thickness*” by Tusan CG *et al*.

Supplementary videos

Supplementary video S1. Colonies on thin hydrogels spread to a greater degree than those on thick materials. MG63 colonies were cultured on soft (1 kPa) Fn coated PA gels. At 2 days post-plating (0 h), the evolution of colonies was recorded for 4 days in a time lapse experiment. On both thin (20 µm) and thick (200 µm) hydrogel, the morphology of colonies was similar at 2 days (0 h), but at later time points, colonies on thin materials spread more than colonies on thick materials.

Supplementary video S2. Cells cultured on thin and soft substrates occasionally remained less associated with the colony, but do not migrate away from the colony and in the end regained contact. MG63 cells were cultured on thin (20 µm) and soft (1 kPa) Fn coated PA hydrogel. At 2 days post-plating (0 h), the evolution of the colony was recorded for 60 h in a time lapse experiment.

Supplementary video S3. Colony induced displacements in the hydrogels. On the top panel is presented the evolution of the MG63 colonies seeded on soft (1 kPa) thin or thick Fn coated PA gels starting with day 2 (0 h). On the bottom panel, it can be seen the displacement induced by the colonies as indicated by the fiducial fluorescent marker beads incorporated in hydrogels.

Supplementary video S4. Colony induced greater displacements in the thick hydrogels. Displacements on thin hydrogels were localised primarily to the regions occupied by cells, whereas on thick hydrogels, displacements extended well beyond the colony periphery. The magnitude of the displacements (see the colour bar) was significantly lower on thin hydrogels compared to thick.

Supplementary video S5. Tracking of gel displacement. Displacements were in general directed inward, radially towards the colony centre, while on thin gels displacements were less directional, with both inward and outward displacements.

Supplementary video S6. The hydrogel deformations induced by the colony consistent with a 'pinching' of the material. The 'pinching' (indicated by the red arrows) was evident visually on fiduciary bead images as an alignment of marker beads in a direction radial to the colony centre.

Supplementary video S7. Coalescence of the colonies. Colonies close in proximity tend to extend towards a neighbouring colony through their pseudopodia. They form connections of cellular bridges between colonies, which subsequently promoted the coalescence of the colonies.

Supplementary figures

FIGURE S1

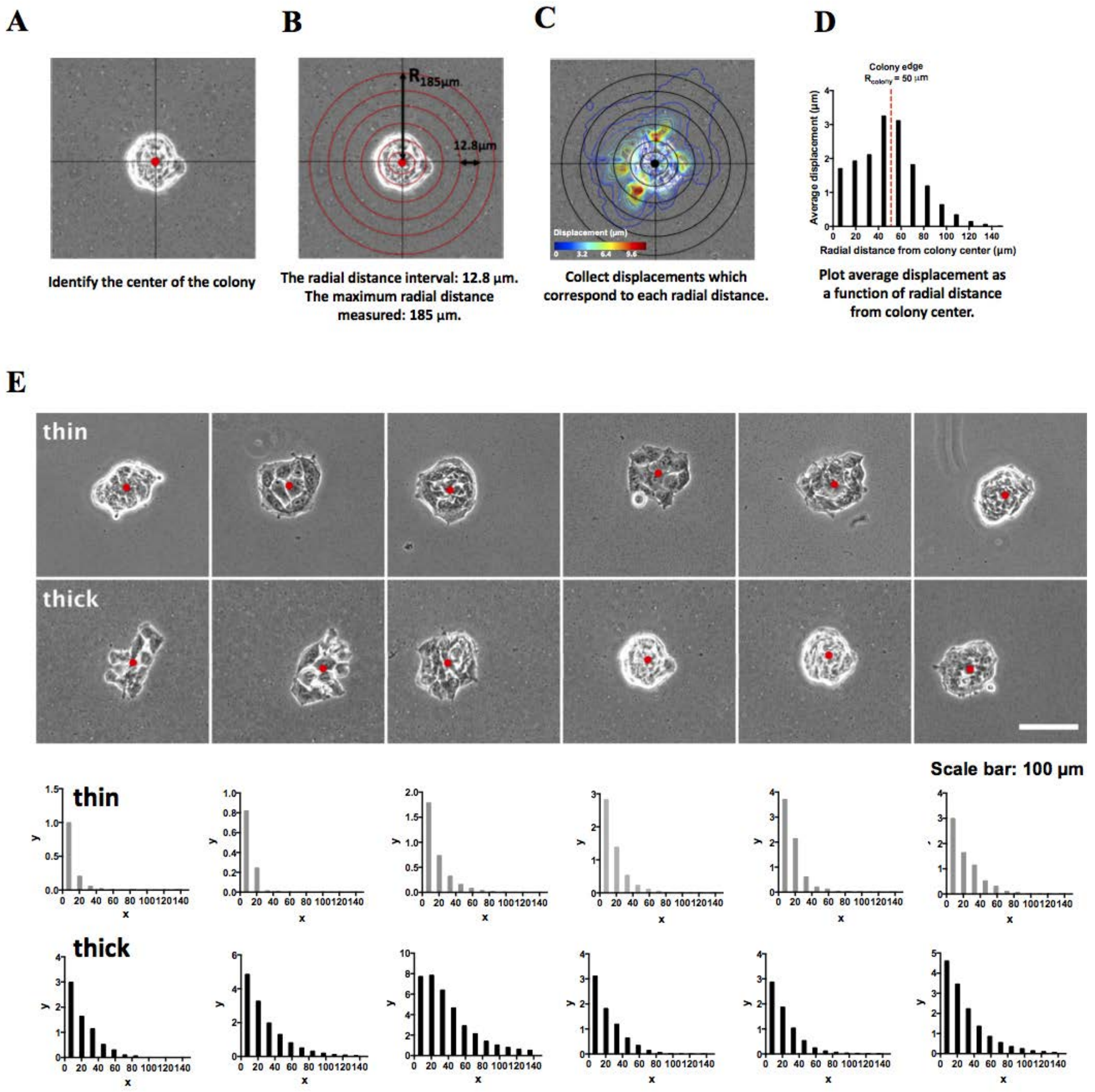

x = Radial distance from colony centre (μm)
y = Average displacement (μm)

Figure S1. Ilustration of the Methods used to calculate the average displacements as a function of the radial distance from the colony edge. The colony centre was determined manually, annotated with a red dot **(A)**. The radial position was divided in equidistant bins from the centre of the colony outwards **(B)**. The nodal displacements were averaged over all nodes in a certain bin **(C)**. The average displacement was plotted as a function of the radial distance from the colony centre. The colony radius was calculated based on the colony area

considered as a perfect circle **(D)**. **(E)** MG63 colonies similar in size (6000-9000 μm^2) cultured on 1kPa Fn coated PA thin and thick hydrogel were analysed. The red dot represents the centre of the colony determined manually. The average displacement as a function of radial distance from the colony edge propagates at greater distances for colonies cultured on thick compare to thin hydrogels.

FIGURE S2

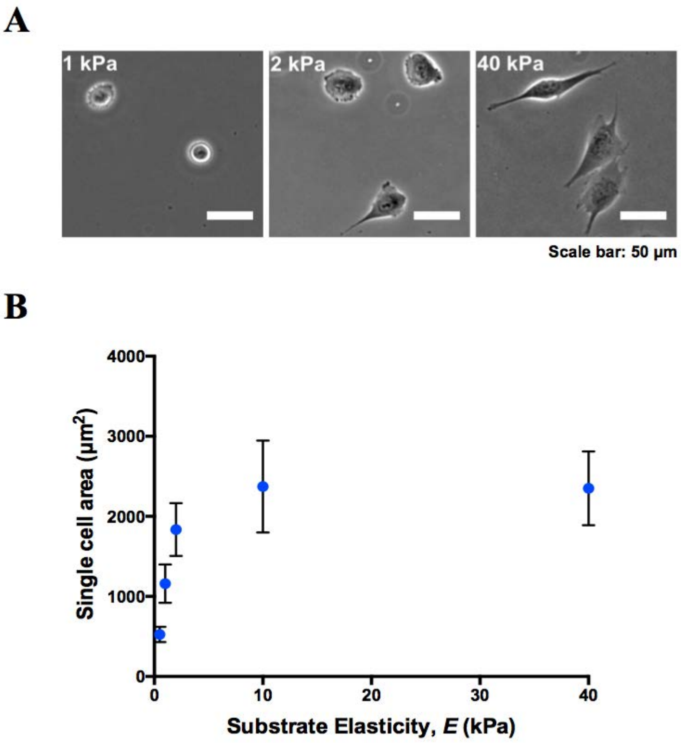

Figure S2. MG63 single cell **area increases with increasing substrate stiffness. (A)** MG63 cells spread to a greater degree on stiff substrates (40 kPa); compared with softer substrates (1 kPa and 2 kPa). **(B)** This phenomenon can be quantified by cell area measurements. Data presented as mean ± SD (n = 10 single cells).

FIGURE S3

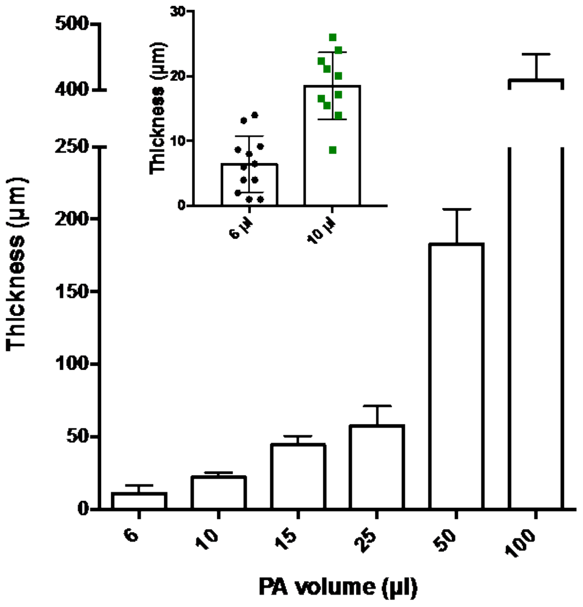

Figure S3. Substrate thickness correlates with acrylamide/bis-acrylamide volume. Substrate thicknesses were measured using the Nikon Eclipse Ti inverted microscope. The thickness of the 1 kPa PA hydrogels increase proportional with the volume used (large graph). The lowest thin hydrogels fabricated was 6.3 ± 4 µm for 6 µL hydrogels and 19.5 ± 5 µm for 10 µL hydrogels (small graph). The error bars represent the ± SD of the mean for the measurements (n = 6) on one substrate.

FIGURE S4

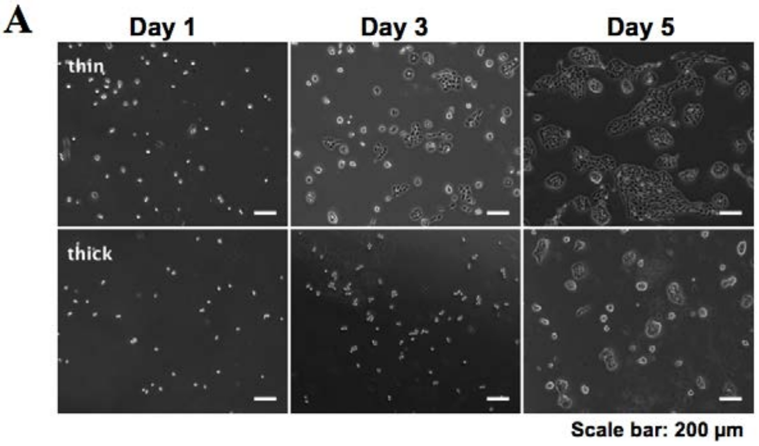

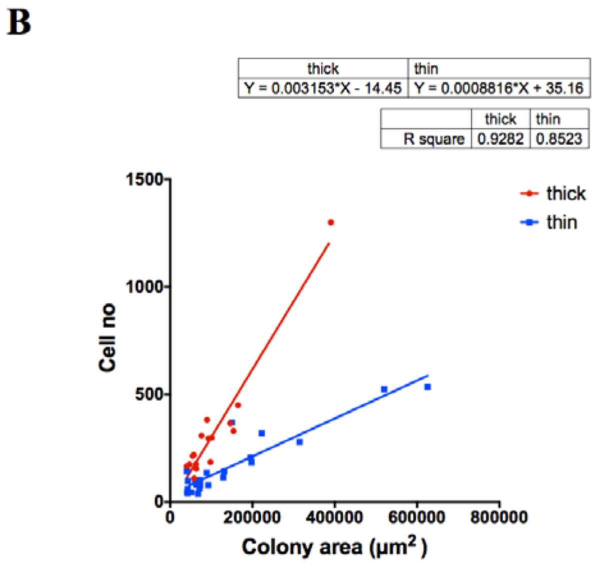

Figure S4. Representative images of MG63 cultured on soft PA hydrogels. (A) MG63 colonies on thin (1 kPa) hydrogel spread to a greater degree and occupy a greater surface area of the hydrogel with respect to those on thick hydrogels, where MG63 cells predominantly remain as small, round and dense colonies. **(B)** On both thin and thick hydrogels, cell number/colony is directly proportional to colony area, but colonies on thick hydrogels are ~4× denser than those on thin.

FIGURE S5

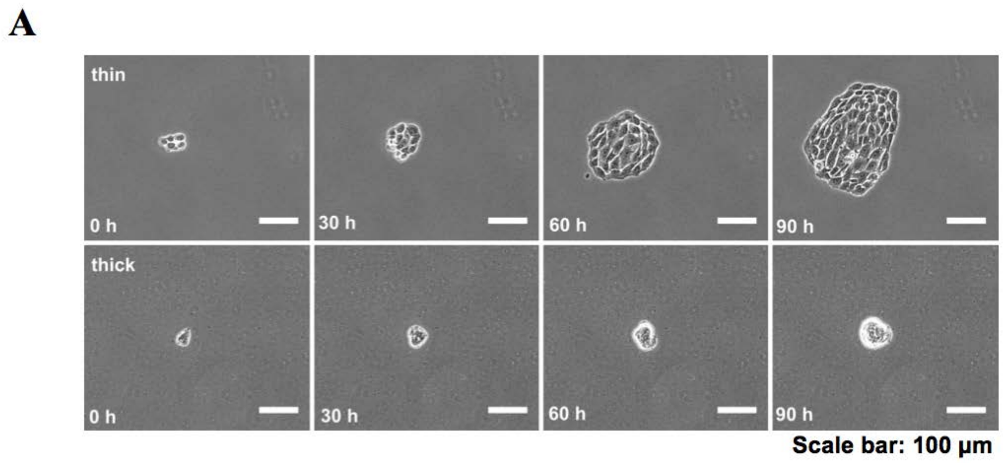

B

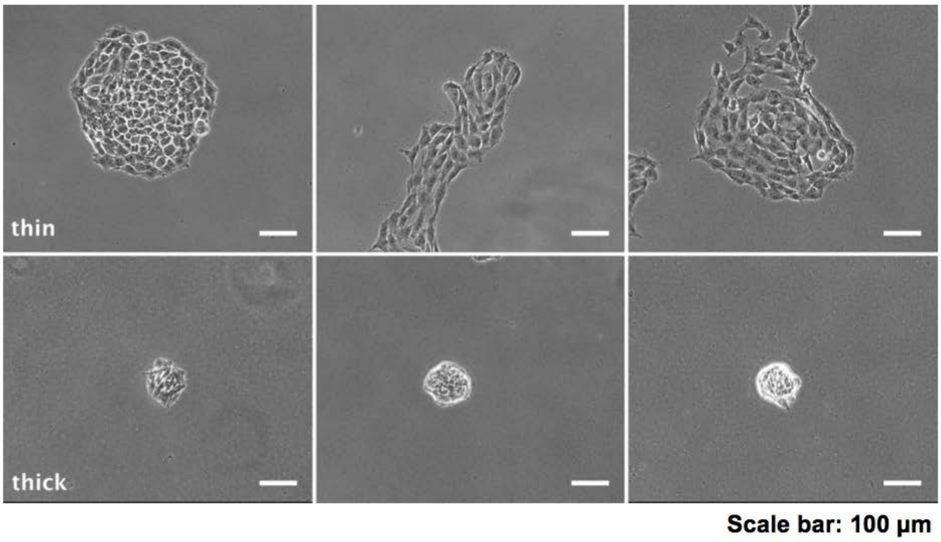

C

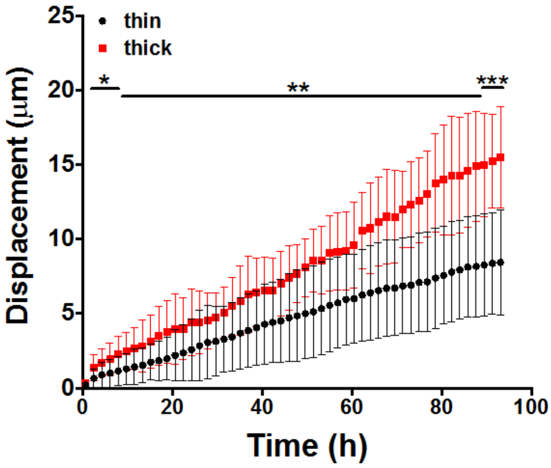

Figure S5. (A) MG63 colonies imaged during a time lapse experiment, from day 2 (t 0h) to day 6 (t 94 h). On thin 1kPa, Fn coated PA hydrogels, MG63 spread to a greater degree compared to those on thick PA hydrogels. Individual flattened cells are visible in the former colony, but not the latter small, round and compact colony. **(B)** Phase contrast images of 3 colonies on thin hydrogels (top) and 3 colonies on thick hydrogels (bottom) at day 6, illustrating the difference in colony morphology **(C)** The highest 10 % of surface displacements exerted by colonies on thin compared to thick hydrogels over the entire 94 h culture period (n=10), increased with respect to time, and it was higher on thick compared to thin hydrogels (significant differences in mean displacement occurred after 10 h in culture (at 94 h, thin: 8.0 ± 3.5 µm, thick 14.8 ± 3.3 µm, ***$p < 0.001$ for 90 – 94 h, **$p < 0.01$ for 10 – 90 h). Data presented as mean ± SD of the colony displacement, n = 10. Statistical significance assessed by Mann-Whitney U test.
